# Prenatal ultrasound exposure and association with postnatal hearing outcomes

**DOI:** 10.1186/1916-0216-42-3

**Published:** 2013-01-31

**Authors:** Claude F Harbarger, Paul M Weinberger, Jack C Borders, Charles A Hughes

**Affiliations:** 1 Department of Otolaryngology / Head and Neck Surgery, Georgia Regents University, 1120 15th Street, BP 4109, Augusta, GA 30912, USA; 2 Department of Surgery-ENT / Maxillo Facial Surgery, Tawam Hospital, Johns Hopkins University, P.O. Box: 15258, Al-Ain, UAE

**Keywords:** Prenatal, Ultrasound, Hearing loss

## Abstract

**Objective:**

Prenatal ultrasound exams have become increasingly frequent. Although no serious adverse effects are known, the public health implications would be enormous should adverse effects on auditory development be shown. This study looks to establish a possible correlation between hearing loss and increased prenatal ultrasound exposure.

**Design:**

Retrospective cohort analysis.

**Setting:**

Tertiary academic referral center.

**Methods:**

A retrospective review of 100 children undergoing newborn hearing screening was conducted. Extensive data collection was performed, and this data was analyzed for a potential correlation between failure of newborn hearing screening and increased prenatal ultrasound exposure, as well as for a potential correlation of other variables with hearing loss.

**Main outcome measures:**

Postnatal hearing outcomes.

**Results:**

A higher number of both total and 3^rd^ trimester ultrasound exams as well as a younger gestational age at birth were all found to be significantly associated with a higher likelihood of passing the newborn hearing screen (p<0.001 for each). No other factors were found to reach statistical significance.

**Conclusions:**

Our results show that there is no correlation between a higher level of prenatal ultrasound exposure and hearing loss. Indeed, infants who had more prenatal ultrasounds in the third trimester were more likely to pass their screening hearing exams. The finding that children receiving more prenatal ultrasounds have a higher likelihood of passing newborn hearing screens serves as an excellent reminder of the classic statistics rule that correlation does not imply causation.

## Introduction

Ultrasonography (US) now has a wide range of clinical uses and has become virtually indispensable in many areas of medicine. The development of prenatal US represents one of the greater achievements in the field of obstetrics. However, despite the widespread prevalence of prenatal US, it is not entirely clear whether US waves are free of risk to the developing fetus. Because of the increasing use of this imaging modality, the public health implications of any potential adverse effects are enormous. These concerns are even more important in light of the fact that routine US screening of the fetus has not been shown to improve perinatal morbidity [1,2], despite an 80% detection rate of fetal anomalies at experienced centers [3].

Only a small number of studies have investigated the immediate and long-term effects of US on fetal development. Links have been proposed between increased US exposure and handedness [4-6], neurological deficits such as speech delay [7], and low birth weight and size [8,9]. Recent studies have also implicated that prolonged exposure to US affects the migration of brain cells in fetal mice [10]. To date in English literature, only one study has looked to assess the potential effect of US on auditory pathway development [11]. Although there was no identified correlation between ultrasound exposed vs. non-ultrasound exposed children and hearing loss, this study was conducted over the years 1968–1972 and thus predates the recent surge in average number of prenatal ultrasound exams. Thus, this study looks to revisit this topic with an analysis of contemporary data.

## Methods

After appropriate Institutional Review Board approval, a retrospective chart review was conducted, using CPT codes for newborn hearing screening over the time period 01/01/2007 to 12/31/2010. Records were reviewed of both children and mothers for fifty children found to have normal (passed) newborn hearing screens and also for 50 children with abnormal (failed) newborn hearing screens.

Hearing screens conducted in the well-newborn nursery were conducted as distortion-product otoacoustic emissions (DPOAE) and those conducted in the neonatal intensive care unit were done as auditory brainstem response (ABR) tests. Data collected included the number of prenatal US exams in each trimester, perinatal and pregnancy complications, gestational age at birth, presence of perinatal hypoxia at birth, maternal substance abuse, maternal age at birth, presence of otologic abnormalities at birth, use of ototoxic medications prenatally or postnatally, comorbidities, birth condition, post-natal course, family history of hearing loss, APGAR scores at 1 and 5 minutes, type and degree of documented hearing loss when available, and length of follow-up. To allow statistical comparison, we classified obstetrical events using the McNeil-Sjostrom Obsterics Complication Scale, which is an established systematic evaluation and weighting of several hundred specific somatic conditions and events during pregnancy, labor-delivery, and the prenatal period [12,13]. We used the American Society of Anesthesiology physical status classification scheme (“ASA grade”) to classify children’s comorbidity status [14]. For statistical analysis, patients were categorized as failing a hearing screen in at least one ear versus passing both ears. Differences between groups were then compared using the non-parametric Wilcoxon Rank-Sums test for each variable assessed. We also performed a logistic regression using forward entry of each variable found to be significant in independent analysis or considered clinically important. Non-parametric Spearman’s correlation was used to assess for co-variance between gestational age and number of US performed in the third trimester. For all tests a p<0.05 was considered significant, and testing was performed using SPSS version 20 (IBM corporation, Armonk, NY).

## Results

Demographics of both the hearing screen failures and control group are summarized in Table 1. Thirty two children in the study group failed the hearing screen in 1 ear, 18 failed in both ears, and the control patients (n=50) passed hearing screens in both ears for a total number of 100 children.

**Table 1:** Patient demographics

**Dependent variable**	**All patients (n=100)**	**Pass both (n=50)**	**Fail at least one ear (n=50)**
GA birth	255.9 (95%CI +/−43.5)	244.66 (95%CI +/− 41.0)	267.08 (95%CI +/−34.2)
US 1st trimester	0.9 (95%CI +/− 2.2)	1.02 (95%CI +/− 2.2)	0.82 (95%CI +/−2.1)
US 2nd trimester	1.84 (95%CI +/− 2.8)	2.10 (95%CI +/− 3.4)	1.58 (95%CI +/−1.9)
US 3rd trimester	5.01 (95%CI +/−7.1)	6.66 (95%CI +/−6.1)	3.36 (95%CI +/−6.6)
US total	7.77 ((95%CI +/−8.9)	9.78 (95%CI +/− 8.1)	5.76 (95%CI +/− 8.0)
McNeil Sjostrom score	2.73 (95%CI +/−)	2.86 (95%CI +/−1.5)	2.60 (95%CI +/− 2.1)
ASA score	1.38 (95%CI +/− 1.3)	1.48 (95%CI +/− 1.4)	1.28 (95%CI +/− 1.6)
Maternal substance abuse	0.14 (95%CI +/− 0.1)	0.14 (95%CI +/− 0.7)	0.14 (95%CI +/− 0.7)
Perinatal hypoxia	0.09 (95%CI +/−0.6)	0.10 (95%CI +/-0.6)	0.08 (95%CI +/− 0.5)
Otologic abnormalities	none	none	none
Familial history of hearing loss	none	none	none
Prenatal ototoxic medication	0.02 (95%CI +/− 0.3)	none	0.04 (95%CI +/− 0.4)
Postnatal ototoxic medication	0.13 (95%CI +/− 0.7)	0.14 (95%CI +/− 0.7)	0.12 (95%CI +/− 0.64)
Apgar 1	7.38 (95%CI +/− 3.7)	6.84 (95%CI +/− 4.3)	7.92 (95%CI +/− 2.6)
Apgar 5	8.28 (95%CI +/− 2.7)	7.92 (95%CI +/− 3.2)	8.64 (95%CI +/− 2.0)
maternal age at birth	27.79 (95%CI +/− 13.0)	27.94 (95%CI +/− 13.1)	27.64 (95%CI +/− 13)

**Figure 1:**
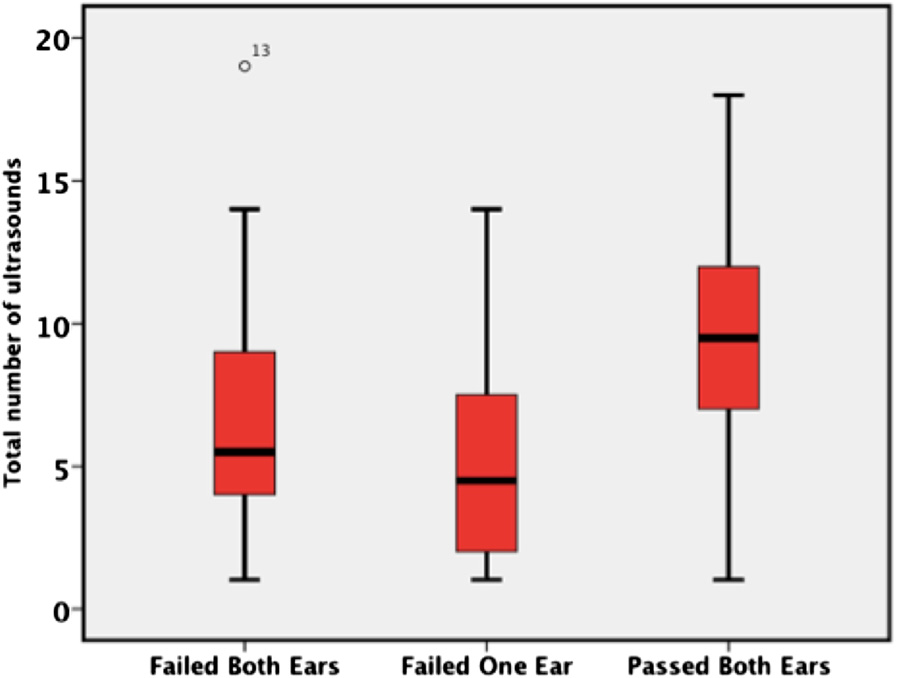
Hearing status and total ultrasound exposure.

**Figure 2:**
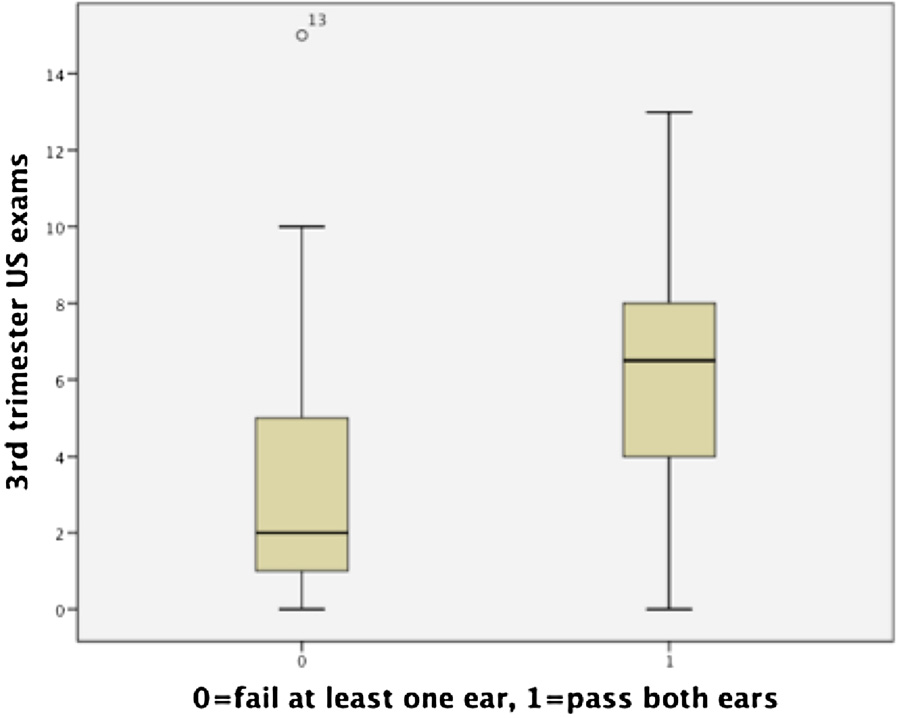
Hearing screen and 3rd trim US exposure.

**Figure 3:**
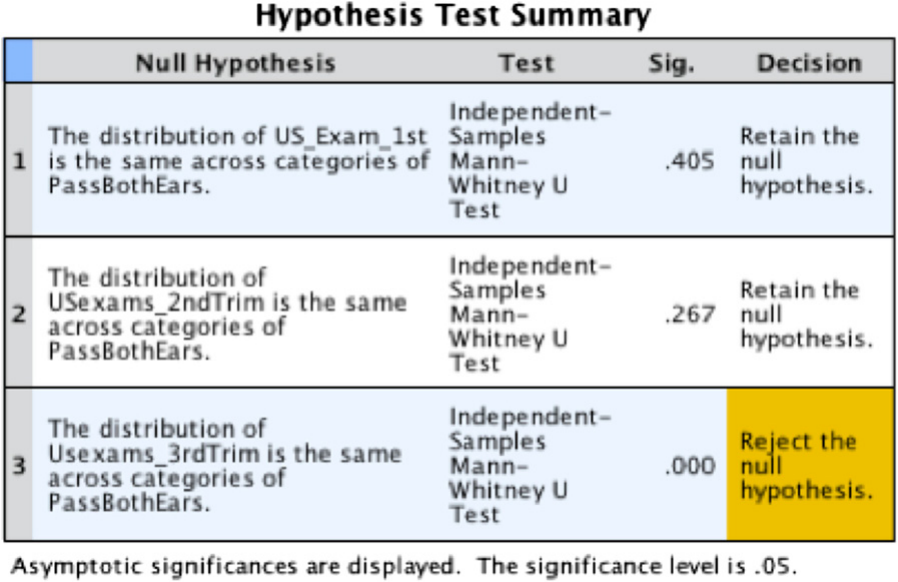
Ultrasound exams per trimester.

**Figure 4:**
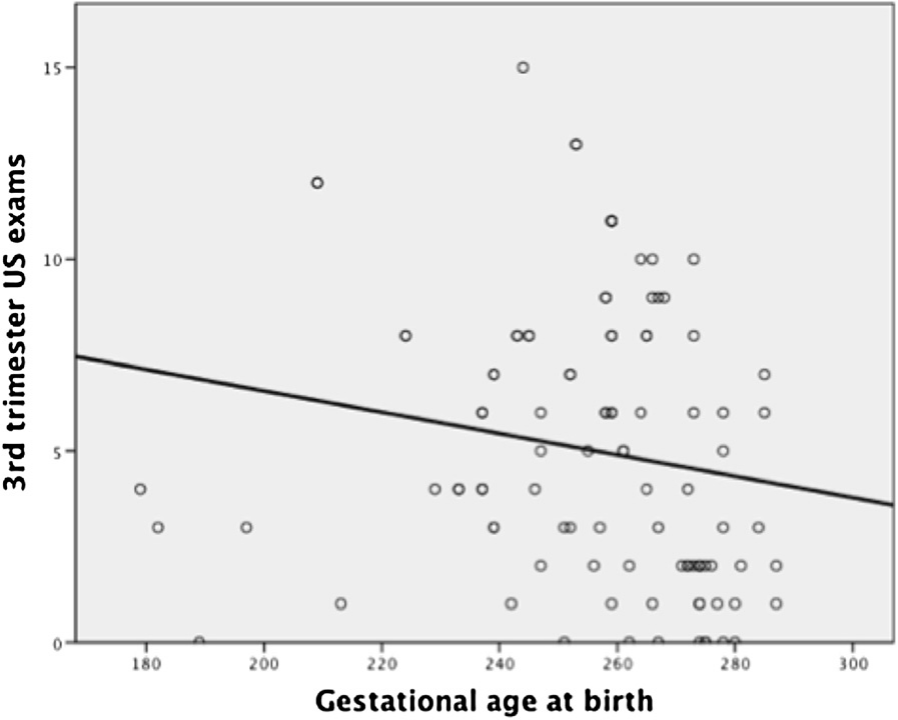
Gestational age and 3rd trimester US exams.

**Figure 5:**
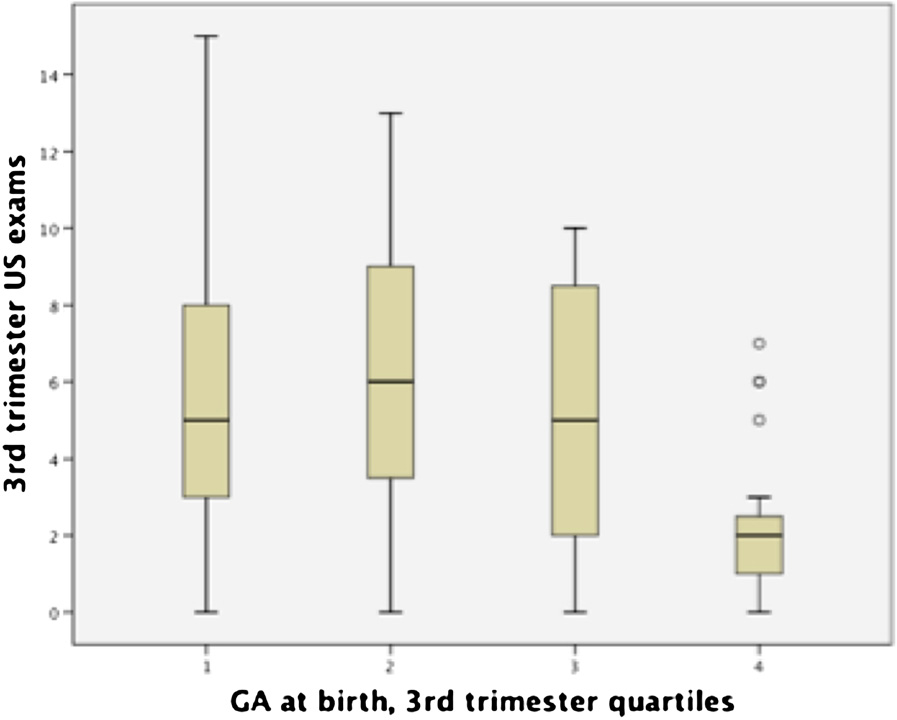
US exams in 3rd trimester.

Using the non-parametric Wilcoxon Rank-Sums test, there was found to be a significant difference in both total number of ultrasounds (p<0.001 respectively) and ultrasounds in the third trimester (p<0.001) between patients with and without failed hearing screens (see Figures 1, 2, 3). Patients who failed screening in at least 1 ear had a median of 5 (inner quartile range 1 to 19) total number of US exams, and 2 (range 0 to 15) exams in the third trimester, compared to 9.5 (range 1–18) and 6.5 (range 0–13) US exams respectively in the control group (Figure 1). Spearman’s correlation found a significant association between gestational age and total number of ultrasounds performed in the third trimester (rho= −0.31, p=0.002, see Figure 4). To further explore this association we divided gestational age into quartiles. As summarized in Figure 5, patients in the highest gestational age quartile (e.g. those born at the latest gestational ages) had fewer ultrasounds compared to the other quartiles.

A binary logistic regression analysis in a stepwise forward fashion showed that both a higher number of 3^rd^ trimester ultrasound exams performed (p=0.01) and a younger gestational age at birth (p<0.001) were significantly associated with a higher likelihood of passing the newborn hearing screen (Table 2). No other factors were found to reach statistical significance.

**Table 2:** Analysis of variables’ association with hearing loss

**Variable**	**Significance**
US exams in 1^st ^trimester	.173
US exams in 2^nd ^trimester	.365
US exams in 3rd trimester	.001
Gestational age at birth	.000
McNeil-Sjostrom score	.821
Perinatal hypoxia	.716
Tobacco	.631
Cannabinoids	.256
Alcohol	.999
Crack	.878
Barbiturates	.889
ASA classification	.951
Ototoxic meds prenatally	.999
Ototoxic meds postnatally	.993
Maternal age at birth	.069
Apgar score at 1 minute	.195
Apgar score at 5 minutes	.748

## Discussion

Diagnostic levels of US have been shown to induce bone formation and have therefore been used therapeutically for a number of years for the treatment of bone fractures [15-17]. US has also been shown to stimulate chondrogenesis of mesenchymal (cartilage) stem cells [18]. Reher et al. [19] found that with diagnostic exposure, osteoblasts produced nitric oxide (NO), a free radical which has been linked to both outer hair cell injury and sensorineural hearing loss [20]. Likewise, other studies have noted increased nitric oxide synthase (NOS) and NO activity in other tissue types following US exposure [21,22]. Indeed, there have been concerns that prenatal US could be associated with sensorineural hearing loss, despite a study by Stark et al. in 1984 that failed to show an association [11]. One recent study found that children with delayed speech were roughly 3 times more likely to have been exposed to prenatal US, although the authors did conclude that it was premature to presume causation [23].

Growing concern over US includes not only the increased prevalence of its use, but also its deregulation in the 1990s [24]. Denser body tissues necessitate higher intensity outputs in order to penetrate deeply enough to produce images of adequate clarity and resolution [25]. With increasing rates of obesity and the consequent need to accommodate US for use on increasing body sizes, the FDA has loosened regulations on the maximum intensity allowed for clinical use.

Despite the above concerns, in the 40 years since introduction into clinical practice, US has not been directly associated with any significant health risk to the fetus or mother [26]. The American Institute of Ultrasound in Medicine has stated that prenatal US should be performed according to the ALARA principle (As Low As Reasonably Achievable), and that it should be performed only with a valid medical indication and with the lowest outputs necessary. Additionally, both the American Institute of Ultrasound in Medicine and the Food and Drug Administration have discouraged the use of US for "keepsake fetal imaging", although this remains prevalent. Additionally, litigation factors may also be driving the increasing use of US. Failure to perform an ultrasound, cardiotocograph or other medical tests at appropriate times is commonly cited in lawsuits against doctors, midwives, and hospitals [27].

Our results show that there is no correlation between a higher level of US exposure prenatally and hearing loss, in fact quite the opposite is true, particularly in the case of third trimester US exams. The otic placode develops during weeks 4–8 of life and inner ear development is complete by the 26^th^ week of gestation. Our presumption was that the inner ear would be most susceptible to ultrasound-induced injury during this developmental period of the 1^st^ and 2^nd^ trimesters. An increased level of ultrasound exposure during this period proved to have no adverse effects on hearing outcomes. Our initial explanation for these findings was that infants born at later gestational age tend to be healthier and more neurologically mature, thus more likely have better hearing outcomes, and that the longer third trimester simply provided an opportunity for more ultrasound examinations. However, with further analysis of the data we found that patients in the highest gestational age quartile had fewer ultrasounds compared to the other quartiles. Thus, we cannot fully explain the better hearing outcomes in the face of increased US exposure, but it is plausible to assume that there is a confounding variable that is associated with improved hearing and more US exams being performed. Although the data show a positive association between number of US exams performed and passing hearing screening in both ears, it is unlikely that US is in and of itself directly protective or beneficial in hearing.

Additionally, we are unable to fully explain our finding that infants passing the newborn hearing screen tended to be younger in comparison to infants that failed. In further analysis of these groups, the only significant difference that was seen was a disproportionately higher number of twins included in the control group that passed. Our initial thought was that twins are generally born earlier in gestational age, but often are healthier in comparison to non-twin hearing screen failures, and thus may have been more likely in comparison to have better hearing outcomes. This did not seem to be supported by further analysis, as twin vs. non-twin comparison found relatively equal distributions in the ASA and McNeil Sjostrom scores across both groups.

One shortcoming of this study is its retrospective nature. Another is that the majority of hearing screens were done as DPOAE’s, which although a reasonable measure of cochlear function, may be negative in the face of a hearing loss of up to 30–40 dB. Thus, our results may have missed a sub-threshold hearing loss that is actually occurring. Additionally, our study had a relatively small *n* and may not have the power to detect a difference if present.

In summary, we found no correlation between a higher level of prenatal ultrasound exposure and hearing loss - in fact quite the opposite is true, particularly in the case of third trimester US exams. Our finding that infants receiving more 3^rd^ trimester ultrasounds have better hearing outcomes serves as an excellent reminder of the classic statistics rule that correlation does not imply causation.

## Consent

This study was reviewed and approved by the Human Assurance Committee of Georgia Health Sciences University (study #Pro00000164). A waiver of informed consent was granted for the study.

## Competing interests

The authors declare that they have no competing interests.

## Authors’ contributions

CFH - Institution Review Board approval process, literature search, data collection, drafted the manuscript. PMW - data interpretation and statistical analysis, CAH - Institution Review Board approval process and manuscript revision JCB - study design and manuscript revision. All authors read and approved the final manuscript.
